# Supplementary material for: Longitudinal Survey of Coronavirus Circulation and Diversity in Insectivorous Bat Colonies in Zimbabwe
Source: Viruses. 2022 Apr 9;14(4):781. doi: 10.3390/v14040781 (PMC9031365; doi:10.3390/v14040781)
Supplement: Supplementary file 1 [file viruses-14-00781-s001.zip › viruses-1620272-supplementary.pdf]

Supplementary materials

Table S1. The reproductive season of dominant bat species across months of the year.

Dominant bat species at both sites	Seasonality	Corresponding months
Hipposideros spp<br>*Rhinolophe spp*<br>*Miniopteris spp*<br>*Nycteris spp*<br>*Macronycteris*	Gestation	01-Sept-2020 to 30-Nov-2020
	Parturition	01-Nov-2020 to 31-Dec-2020
	Lactation	01-Nov -2020 to 31-Jan-2021
	Weaning	01-Feb-2021 to 28-Feb-2021
	4-6 months juveniles	01-Mar-2021 to 31-May-2021
	Non-gestation	01-June-2021 to 31-Jul-2021

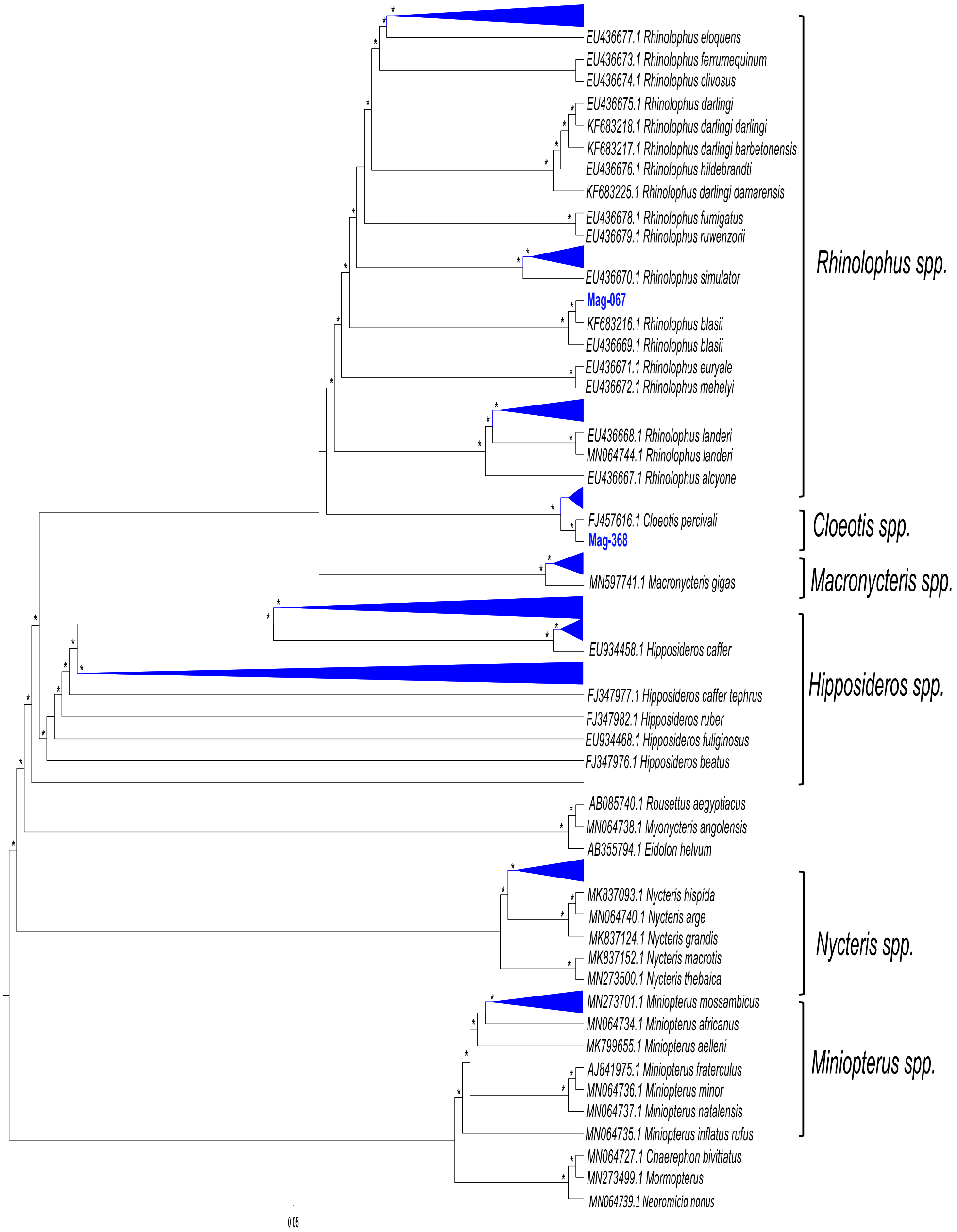

Figure S1. Phylogenetic tree of bat species identified by Cytochrome b. The sequences detected in Zimbabwe are marked by blue colour. The tree was constructed using the maximum likelihood method based on the GTR + F+ I + G4 model. The robustness of nodes was assessed with 1000 bootstrap replicates. Bootstrap values >70 are in asterisk and those <70 are not shown.

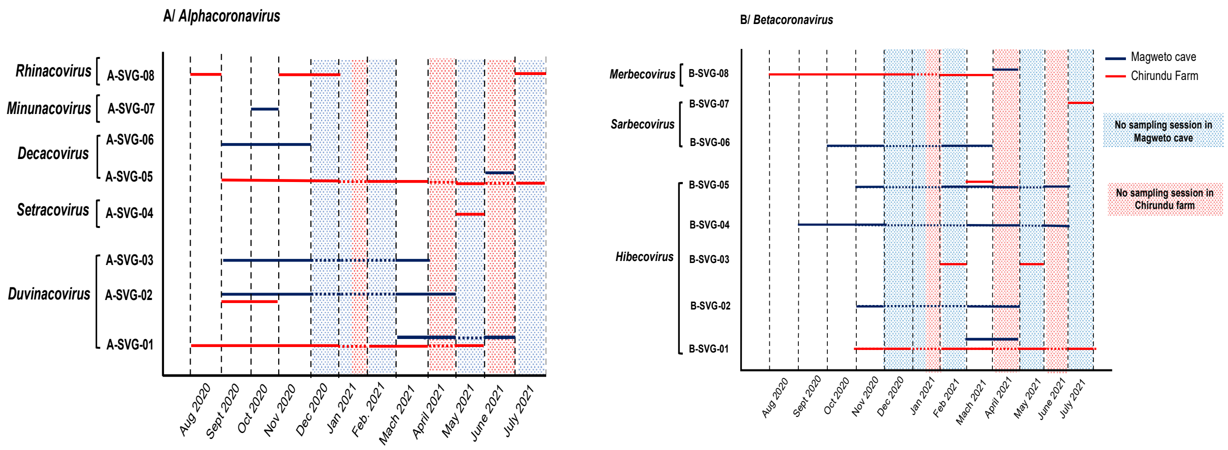

Figure S2. Timeline of sub-viral groups detection at Chirundu and Magweto sites for: (**A**) *Alphacorona-virus*, (**B**) *Betacoronavirus*. Chirundu site is in red colour and Magweto in blue.

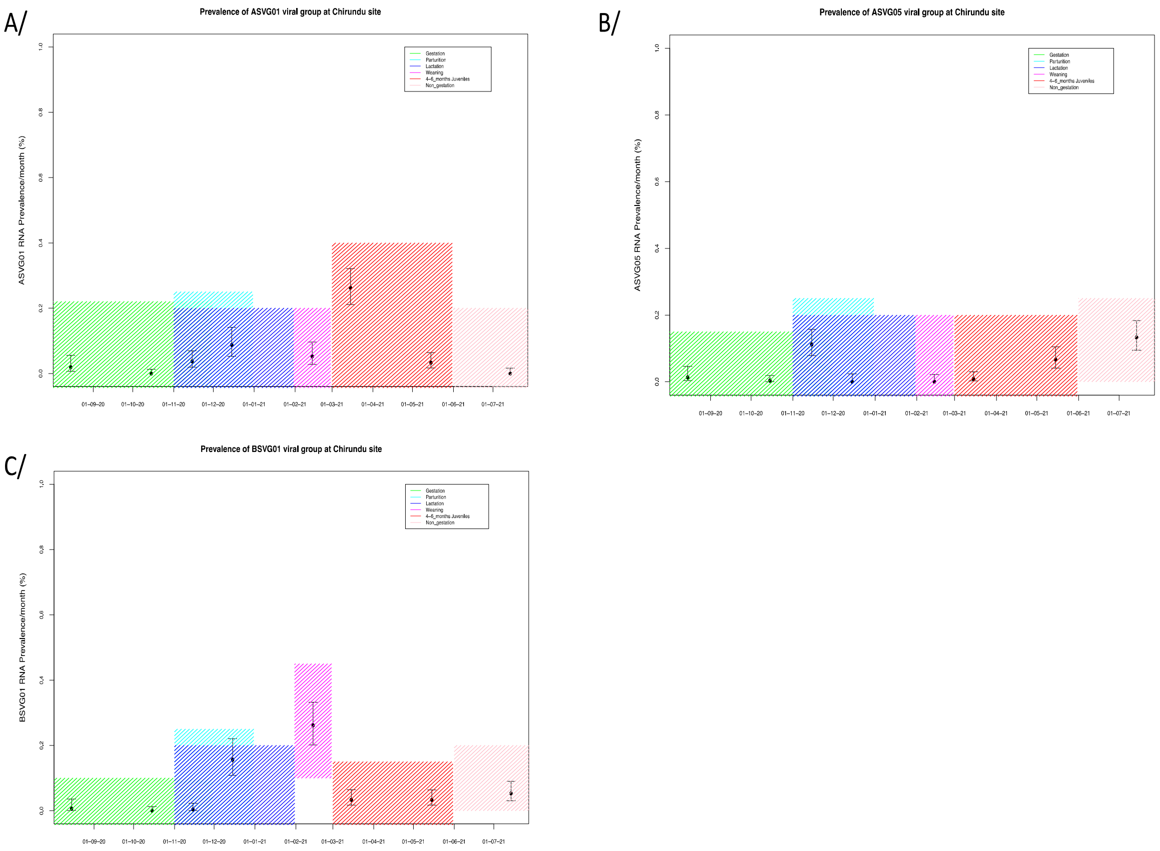

Figure S3. Results of the Chirundu site sub-viral groups CoVs prevalence. The figure shows estimation of the coronavirus prevalence (with CI 95%) with (**A**) A-SVG-01 RNA CoV prevalence, (**B**) A-SVG-05 RNA CoV prevalence and (**C**) B-SVG-01 RNA CoV prevalence. The areas coloured green, cyan, navy blue, magenta, red and pink show periods of gestation, parturition, lactation, weaning, 4-6 months old juvenile and non-gestation periods respectively observed in the dominant bat species at both sites.
